# Elucidating the Aetiology of Human *Campylobacter coli* Infections

**DOI:** 10.1371/journal.pone.0064504

**Published:** 2013-05-29

**Authors:** Francois Roux, Emma Sproston, Ovidiu Rotariu, Marion MacRae, Samuel K. Sheppard, Paul Bessell, Alison Smith-Palmer, John Cowden, Martin C. J. Maiden, Ken J. Forbes, Norval J. C. Strachan

**Affiliations:** 1 School of Biological Sciences, University of Aberdeen, Aberdeen, United Kingdom; 2 School of Medicine and Dentistry, University of Aberdeen, Aberdeen, United Kingdom; 3 Medical Microbiology and Infectious Diseases, College of Medicine, Swansea University, Swansea, United Kingdom; 4 The Roslin Institute, University of Edinburgh, Edinburgh, United Kingdom; 5 Health Protection Scotland, National Services Scotland, Glasgow, United Kingdom; 6 Department of Zoology, University of Oxford, Oxford, United Kingdom; Free University of Berlin, Germany

## Abstract

There has been little research on the determinants of *Campylobacter coli* infection, despite its contributing up to 10% of human *Campylobacter* infections. A case-control and two case-case study methods explored the aetiology of *C. coli* over a one year period across Scotland. The case-control multivariate model found an increased risk of *C. coli* infection in people older than 19 years (O.R. = 3.352), and during the summer months (O.R. = 2.596), while residing in an urban area decreased the risk (O.R. = 0.546). The first case-case study compared *C. coli* and *C. jejuni* cases and also showed a higher risk of *C. coli* during the summer (O.R. = 1.313) and in people older than 19 years (O.R. = 0.791). Living in an urban area was associated with a reduced risk of infection (O.R. = 0.769). Multi-locus sequence typing (MLST) indicated that sheep and chicken *C. coli* sequence types (STs) were most frequently found in humans whilst those from cattle and pigs were rarer. MLST diversity was high in isolates from pigs and chicken, intermediate in human isolates, and low in ruminant isolates. The second case-case study used MLST data to ascribe putative sources of infection to the cases. The putative source for 40% of cases was chicken, with 60% acquired from other sources (ruminants 54% and pigs 6%). The case-case analysis also showed that female gender was a risk factor (O.R. = 1.940), which may be explained by females being more likely to prepare poultry in the home. These findings indicate differences between the aetiology of *C. coli* and *C. jejuni* infections: this should be taken into account by public health professionals when developing strategies to reduce the burden of human campylobacteriosis.

## Introduction

Human campylobacteriosis is the most commonly reported bacterial gastrointestinal infectious disease in the world [1], [2] with an estimated 572,000 community cases in the UK during 2009 [3] and 845,000 cases in the USA annually [4]. *Campylobacter jejuni* and *Campylobacter coli* are the commonest species to cause human infections, with approximately 9% of human infections being caused by *C. coli* in the USA [5] and approximately 7% in England and Wales [6]. Consequently most research has concentrated on the epidemiology of *C. jejuni*, and there is a more limited understanding of the aetiology of human *C. coli* infections [7].

The symptoms of human campylobacteriosis include diarrhoea (which can be bloody), abdominal pain and fever [8]. About 10% of reported cases are hospitalised [9] and, although rare, severe sequelae include Guillain-Barré syndrome, arthritis, or gastrointestinal perforation and occasionally death [8], [10]. In England and Wales the symptoms caused by *C. jejuni* and *C. coli* appear to be clinically indistinguishable, [6] however in the Netherlands diarrhoea is reported in fewer cases of *C. coli* than *C. jejuni* [11].

*C. jejuni* and *C. coli* are zoonoses and both species are frequently carried asymptomatically in a wide range of domesticated livestock (cattle, sheep, pigs, chickens, and turkeys) and wildlife (birds, voles, insects etc.) [12]. They can also be found in symptomatic cats and dogs [13]. Pigs usually have a higher prevalence of *C. coli* than *C. jejuni* [14], [15] whilst most other animals tend to carry a higher proportion of *C. jejuni* (e.g.>65% for poultry, sheep, cattle and wild birds [15]). Most human *Campylobacter* infections are sporadic and outbreaks are rare [16]. The vehicles of infection in recognised household and community *Campylobacter* spp. outbreaks include contaminated water, unpasteurized milk, and chicken liver pâté [17].

Case-control studies have been conducted on sporadic campylobacter cases (*C. jejuni* and *C. coli* combined or *C. jejuni* alone). The main source of infection identified in these studies is fresh chicken, including both the handling of raw and consumption of undercooked chicken [18], [19]. Environmental sources (e.g. contaminated water), contact with domesticated and wild animals and recent travel (particularly foreign) are also important in some settings [2], [20]–[22]. However, at most only half of all cases are explained in the majority of studies, and the only published case-control study of *C. coli* involved small numbers of cases (121) [11].

A case-case methodology [6] identified differences in risk factors between the two species, where cases of *C. coli* infection were more likely to drink bottled water, eat pâté, and tended on average to be older than *C. jejuni* cases. Cases of *C. jejuni* infection were more likely to have had contact with farm animals, and develop illness during the summer months. The case-case methodology minimizes a number of possible biases inherent in case-control studies that include representativeness of reporting in the health care system. However, it is worth noting that the *C. jejuni* case controls are not representative of the population as a whole and hence it is not possible to extrapolate the results to the general population [23].

The *Campylobacter* genome is highly variable and frequent recombination complicates the typing of isolates. The advent of sequence-based typing methods, in particular multi locus sequence typing (MLST) [24], has helped both the characterisation of isolates and provided evidence of host association (i.e. strains that are more commonly found from a particular animal reservoir). MLST has the advantage of being unambiguous, reproducible, and portable allowing rapid exchange of data between laboratories and the creation of reference databases (e.g. PubMLST www.pubmlst.org/campylobacter). Source attribution has employed MLST data to identify the putative origin of combined *C. jejuni* and *C. coli* clinical isolates with poultry being identified as the main source for *C. jejuni.* Poultry and sheep were the main source species for *C. coli* [25]. MLST-based source attribution has also been combined with risk factor analysis for *C. jejuni* in a case-case study that compared ruminant and poultry types [26]. It was found that women were at greater risk of infection from poultry types and it was hypothesised that this was because they were involved in preparation of chicken in the home. In the Netherlands [18] a case-control study combined MLST source attribution data with risk factors. These researchers reported that chicken and ruminant associated genotypes only partially explained foodborne transmission and that it was likely that environmental transmission (i.e. following contact with a contaminated environment) was also important. No studies have previously been performed that combine case-case and case control studies solely on *C. coli* using genotyping data.

Scotland, with a population of 5.25 million, is an appropriate area to conduct investigations into the aetiology of human *C. coli* infection because of its relatively high disease incidence (approximately 95 cases per 100,000 [13], its spectrum of demographic (e.g. rural and urban) and social (e.g. affluent and deprived) characteristics and the wide range of risk factors to which its population is exposed. The aim of this paper is investigate the aetiology of human *C. coli* infections using genotyped isolates by conducting and analysing (1) a simulated case-control study where Scottish *C. coli* cases are compared to randomly generated controls from the human population, (2) a case-case study that compares *C. coli* cases to *C. jejuni* cases, (3) comparing MLST genotypes from humans and animals to determine their genealogy, source attribution and diversity and (4) a case-case study that compares human *C. coli* cases attributed to chicken with those assigned to other animal reservoirs.

## Materials and Methods

### Data

A clinical dataset comprising 2,733 *C. jejuni* and 307 *C. coli* cases typed by MLST was collected from across Scotland from 1^st^ September 2005 to 31^st^ August 2006. This comprised 52% of the total reported Scottish cases over this period. Case information was anonymous but included the postcode sector of main residence, age, gender, and the date of the laboratory report [13] (See File S1). Human population data stratified by age, postcode sector and gender was obtained from the 2001 Scottish census. The Carstairs index of deprivation was used to describe the socioeconomic status of the human population [27]. Cattle, pig, sheep and poultry numbers in 2 by 2 km tetrads were obtained from the 2004 Scottish agricultural census and these were integrated into postcode sectors using ArcView 3.3 (ESRI, Redlands, California, USA).

### Risk Factors for Case-control and Case-case Analysis

Six parameters were available as putative risk factors; (1) age (young - 0−19 yrs old and adult - >20 yrs old), (2) gender (male or female), (3) season (summer - June to August - or the rest of the year), (4) rural or urban human population density (rural - <200 individuals/km^2^, urban - ≥200 individuals/km^2^), (5) deprived (Carstairs index≥0) or affluent (Carstairs index <0) and (6) animal population density. The animal population density (cattle, pigs, poultry and sheep) were subdivided into four groups: group 1 (null density), group 2 (low density), group 3 (medium density), group 4 (high density) for each postcode sector (see File S1).

All of the predictive variables were used in three observational analyses employing univariate and multivariate logistic regression employing the epidemiological modelling software package EGRET (EGRET, version 2.0.3, Cytel Software Corporation, Cambridge, MA, USA). Results for each risk factor were considered as statistically significant when P<0.05. Factors from the univariate analyses with a P value of <0.25 were used in the multivariate analysis.

### Case-control

This analysis compared the 307 *C. coli* clinical cases with 921 controls generated by randomly sampling the human population as described by the Scottish census (www.scrol.gov.uk).

### Case-case *C. coli* versus *C. jejuni*

307 *C. coli* cases were compared with the 2,733 *C. jejuni* cases as controls.

### MLST Analysis

The clonal genealogy of *C. coli* sequence types (STs) was estimated using a model-based approach for determining bacterial microevolution: ClonalFrame software (version 1.0; http://www2.warwick.ac.uk/fac/sci/statistics/staff/research/didelot/clonalframe/ [28]. This approach incorporates both point mutation and recombination events. The program was run with 50,000 “burn-in” iterations which are discarded to minimise the effects of initial values followed by 50,000 data collection iterations. The consensus trees represent combined data from three independent runs, with 75% consensus required for inference of relatedness. The probable reservoir origin of *C. coli* MLST sequence types (STs) was investigated by STRUCTURE genetic population software [29]. Using this method, STs can be probabilistically assigned to ancestral populations based on their frequency. A source dataset of *C. coli* strains with known origins was used as a source reference population and clinical isolates were attributed to this based on ST similarities (See File S2). This source dataset comprised 85 cattle, 322 pigs, 459 chicken and 57 sheep isolates (see File S3) obtained from both the PubMLST database and the CaMPS study [13]. The diversity of cases was determined by Simpson’s index [30] where a value of 0 indicates homogenous STs and a value of 1indicates a heterogeneous population with maximum diversity. Confidence intervals were calculated using a bootstrap method from the PopTools add-in for Microsoft Excel (available from http://www.cse.csiro.au/poptools).

### Case-case Chicken Attributed Strains versus Non Chicken Strains

The *C. coli* STs from cases were assigned to putative source (chicken or non-chicken - cattle, pigs and sheep) when the attribution score was greater than 0.6 (See Files S3 and S4). This analysis then compared 113 *C. coli* cases attributed to chicken with the 181 non chicken cases as controls. Scores from 13 cases were too ambiguous to determine source and were removed from this further analysis.

### Ethics Statement

‘The Multi-Centre Research Ethics Committee (MREC) for Scotland granted an ethical approval (REC ref: 06/MRE00/85) for acquisition and use of the dataset; additionally, approval for the research was obtained from the Research and Development Committee in each of the NHS Health Boards.

## Results

### Case-control Study

In univariate analysis *C. coli* cases were more common in adults than children, in rural rather than urban environments, in affluent as opposed to deprived areas, in postcode sectors with a high pig density and during the summer compared to the remainder of the year (Table 1). These were the only statistically significant factors used in the multivariate analysis as none of the rest had P values <0.25. The multivariate analysis also found that human campylobacteriosis from *C. coli* was statistically significantly associated with being an adult, living in a rural area, and contracting the disease during the summer months.

**Table 1:** Results of the logistic regression for the case-control study.

Factors	Unit	O.R.	C.I. (95%)	P-value
(A) Univariate				
Age	child	1	–	–
	adult	3.346	2.234–5.013	0.000*†
Gender	male	1	–	–
	female	0.878	0.678–1.137	0.323
Season	rest of year	1	–	–
	summer	2.531	1.935–3.311	0.000*†
Location	rural	1	–	–
	urban	0.573	0.437–0.751	0.000*†
Carstairs	affluent	1	–	–
	deprived	0.654	0.502–0.851	0.002*†
cattle density^a^	low density	1	–	–
	high density	0.985	0.880–1.103	0.796
pig density^a^	low density	1	–	–
	high density	1.167	1.050–1.298	0.004*†
poultry density^a^	low density	1	–	–
	high density	1.034	0.924–1.157	0.557
sheep density^a^	low density	1	–	–
	high density	1.023	0.911–1.149	0.701
(B) Multivariate				
age	child	1	–	–
	adult	3.352	2.221–5.059	0.000*
season	rest of year	1	–	–
	summer	2.596	1.969–3.423	0.000*
location	rural	1	–	–
	urban	0.546	0.411–0.724	0.000*

(A) Odd ratios and their associated p-value for all the selected cases in the univariate models. Factors with P<0.05 are considered as significant (*). Factors with a P<0.25 are entered in the multivariate model (†).

(B) Odd ratios and P-values for the final multivariate model. Previous steps, consisting in removing one by one the factors with the highest P-value at each step, are not shown. The program used to execute the analysis gave P = 0.0000 for the overall model fit equal to 0.0000.

a Animals are grouped into four density groups (see File S1) and the odds ratio indicates the relative amount by which the odds of the outcome changes when the value of the predictor value is increased by 1.0 unit.

### Case-case Studies and MLST Analysis

The first case-case analysis comparing *C. coli* cases to those from *C. jejuni* found that *C. coli* cases were more frequent in adults and during the summer months (Table 2). Only one other factor – residence in a rural area - had P<0.25, and was added to the multivariate model. The multivariate analysis showed the same pattern with an increased probability of *C. coli* infection in adults, living in a rural areas and during the summer.

**Table 2:** Results of the logistic regression for the case-case studies.

		*C. coli* (cases) versus *C. jejuni* (controls)					Chicken (cases) versus non chicken (controls)		
Factors	Reference	O.R.	C.I. (95%)	P-value			O.R.	C.I. (95%)	P-value
(A)Univariate									
age	child	1	–	–			1	–	–
	adult	1.696	1.147–2.506	0.008*†			0.816	0.371–1.795	0.614
gender	male	1	–	–			1	–	–
	female	1.091	0.862–1.382	0.469			1.940	1.205–3.125	0.006*†
season	rest of year	1	–	–			1	–	–
	summer	1.285	1.014–1.628	0.038*†			1.362	0.850–2.182	0.200†
location	rural	1	–	–			1	–	–
	urban	0.793	0.622–1.010	0.060†			1.143	0.705–1.853	0.589
Carstairs	affluent	1	–	–			1	–	–
	deprived	1.021	0.801–1.301	0.866			0.830	0.510–1.350	0.452
cattle density^a^	low density	1	–	–			1	–	–
	high density	0.962	0.867–1.069	0.473			1.056	0.860–1.296	0.604
pig density^a^	low density	1	–	–			1	–	–
	high density	0.975	0.888–1.071	0.597			1.107	0.918–1.336	0.287
poultry density^a^	low density	1	–	–			1	–	–
	high density	0.969	0.876–1.071	0.533			0.999	0.816–1.222	0.991
sheep density^a^	low density	1	–	–			1	–	–
	high density	1.026	0.921–1.144	0.643			1.017	0.827–1.251	0.874
(B) Multivariate									
gender	child	1	–	–	gender	male	1	–	–
	adult	1.791	1.209–2.653	0.004*		female	1.940	1.205–3.125	0.006*
season	rest of year	1	–	–					
	summer	1.313	1.035–1.665	0.025*					
location	rural	1	–	–					
	urban	0.769	0.603–0.981	0.034*					

(A) Odd ratios and their associated P–value for all the selected cases in the univariate models. Factors with P<0.05 are considered as significant (*). Factors with a P<0.25 are entered in the multivariate model (†).

(B) Odd ratios and p-values for the final multivariate models. Previous steps, consisting in removing one by one the factors with the highest p-Value at each step, are not shown. The program used to execute the analysis gave P = 0.0060 for the overall model fit for the chicken versus non chicken case-case study, and P = 0.0006 for the *C. coli* versus *C. jejuni* case-case study. Because gender is the only factor kept at the end of the multivariate model in the chicken versus non chicken study, odd ratio and P-Value are the same as in the univariate gender model.

a Animals are grouped into four density groups (see File S1) and the odds ratio indicates the relative amount by which the odds of the outcome changes when the value of the predictor value is increased by 1.0 unit.

**Figure 1:**
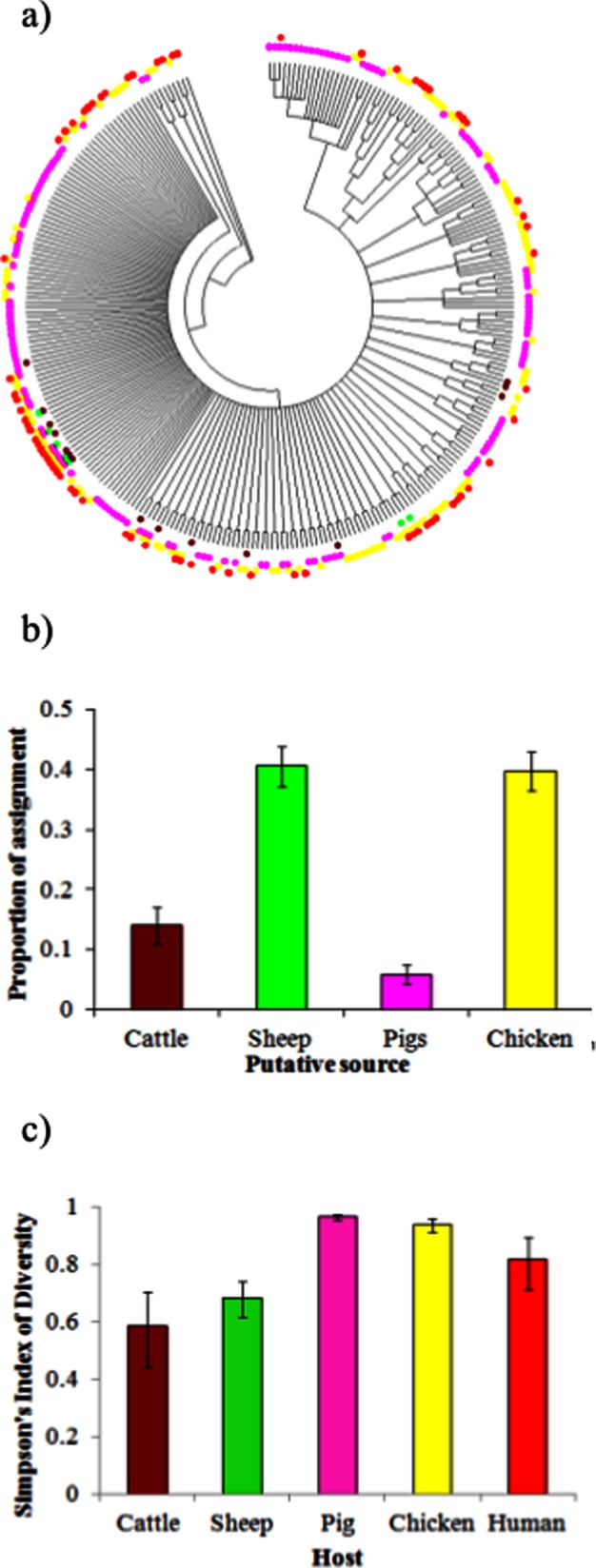
A, ClonalFrame tree of *C. coli* by host (brown – cattle, green – sheep, pink – pigs, yellow – chicken and red – human clinical. B, probabilistic assignment of the host of human *C. coli* infections using structure attribution model (four equal sized columns would be expected in the absence of any genetic differentiation by host). C, Simpson’s index of diversity by host.

The ClonalFrame phylogeny of *C. coli* sequence types (Fig. 1A) shows that particular clades dominated particular hosts. It was observed that 31% of cattle, 100% of sheep, 17% of pig and 62% of chicken ST’s are also found in humans. Attribution by structure (Fig. 1B) assigns 41% of human clinical cases to sheep, 40% to chicken and lower proportions to cattle (14%) and pigs (66%). Simpson’s index (Fig. 1C) shows that pigs and chickens have the greatest diversity of *C. coli* ST’s, whilst cattle and sheep the least with humans being intermediate.

The univariate case-case analysis comparing chicken attributed STs to non-chicken STs (Table 2) showed females more likely to be infected than males to be infected by chicken strains. Similarly, in the multivariate analysis where only gender and season were used in the analysis (P<0.25), only gender was statistically significant (P = 0.006), supporting the observation that *C. coli* infections involving strains attributed to chicken were more common in females.

## Discussion

The case-case (*C. coli*- *C. jejuni*) study shows that there are a higher proportion of *C. coli* than *C. jejuni* cases in adults than children. This finding has been reported previously [6], [13], [31] where it was found that *C. coli* incidence is higher in older than younger people. The reasons for this are unknown although it is likely to be due to behavioural factors, influencing exposure, or physiological factors, influencing susceptibility, or a combination of both. One possibility is differential acid resistance between *C. coli* and *C. jejuni*. This would have the greatest impact in the adult/elderly population, where proton pump inhibitors are more heavily used and have been demonstrated to be associated with increased risk of campylobacteriosis [10]. The seasonality of human campylobacteriosis has been researched extensively, although this has been primarily on all *Campylobacter* infections [32], [33]. The case-control study indicates that there is a higher incidence of *C. coli* infection in the summer months and this can potentially be explained by the same risk factors associated with increased *C. jejuni* infection in summer (e.g. travel, greater exposure to environmental sources, greater prevalence in poultry resulting in increased human exposure and therefore infection). Further work is required to establish which of these factors are the most relevant, by attributing seasonal *C. coli* cases to source. However, the case-case finding that *C. coli* infection has a higher summer incidence than that for *C. jejuni* which differs from results previously published from England and Wales [6]. The reasons for this are unclear. Most of the poultry consumed within the UK is farmed, processed and distributed within the country, so differences in farming or production between Scotland and the rest of the UK is unlikely to provide an explanation. Travel, particularly abroad, is likely to be more common across the UK during the summer months. England has easier access to the continent than Scotland does, and has a higher proportion of first and second generation immigrants who may be more likely to travel abroad to meet family etc. [6]. Again, further work is required to understand the difference of increased *C. coli* incidence during the summer months.

The decreased risk of *C. coli* infection in urban areas reported by the case-control study is likely to be due either to greater environmental exposure in rural areas or a reporting bias. Consumption rates of poultry have been reported to be the same in rural and urban populations [34]. However, the case-control study failed to find any association between *C. coli* infection and farm animal densities (the main environmental reservoir) except for pigs. A case-control study in the Netherlands [11] found an increased *C. coli* incidence in urban areas which contradicts our findings. Other more proximate risk factors could be investigated (e.g. being on a private water supply, direct contact with farm animals etc.), which may help to explain this finding. Pigs are a potential source of human *C. coli* infection because they have a high prevalence (e.g. up to 99% [14]), especially when compared to other sources. However source attribution suggests that they are relatively unimportant, with only 6% of human *C. coli* cases being attributed to pigs. There is a high diversity of genotypes observed in pigs and only a small proportion of these STs (17%) were associated with human illness in the current study. Prevalence rates on retail pork are low (<0.5%) compared to those found on retail chicken (52–90%) [35], [36] which suggests the foodborne route is not a major contributing factor. The univariate case-control study showed a significant association of *C. coli* infection with increasing pig density. However, the odds ratio being close to one (O.R. = 1.167) suggests that pig density can only potentially explain a small proportion of the actual number of cases. The univariate analysis also found that there was a greater risk of *C. coli* infection for cases living in more affluent areas and this has already been reported elsewhere for *Campylobacter* spp. infections [37]. It remains unclear whether this effect is real or associated with a reporting artefact [38]. In the multivariate model, affluence is no longer statistically significant and it is likely that this is correlated to some extent with population density since the poorest areas in Scotland are generally located in postal sectors with the highest population density.

The case-case study utilising source attribution data found that female gender was associated with chicken STs. This agrees with a similar study for *C. jejuni* [26]. It is unknown whether this difference is a behavioural or physiological phenomenon: a possible explanation is that women are more likely to handle and prepare raw chicken in the home and as a consequence are at a greater risk. This is corroborated by research findings in the USA which report that on average women spend three times longer than men preparing food each week [39]. However, it is also possible that increased female susceptibility plays a role [40].

The source attribution analysis indicated that both sheep (41%) and chicken (40%) were the main sources of human *C. coli* infection. This is different to source attribution studies for *C. jejuni* in Scotland, North-West England [41], New Zealand [42] and The Netherlands [18] which all showed that chicken had a higher attribution to human cases (57–80%) than sheep (2.5–24%). This raises the question of why sheep are potentially as important for *C. coli* infection as chicken in Scotland. Further, which pathways (e.g. foodborne or environmental - e.g. direct contact with the environment or waterborne) are most important? Retail surveys of chicken [43], [44] show that approximately 30% are contaminated with *C. coli* whereas *C. coli* (and *C. jejuni*) are rarely isolated from red meats e.g. *C. coli* was absent in 1,056 retail lamb samples from the UK [35]. In addition the consumption of chicken is higher than that of sheep products [45]. This suggests that the foodborne route for human *C. coli* infection is more likely to be associated with chicken, which is counter to the source attribution findings. When considering environmental transmission to humans in rural areas, human contact through animal faeces, either directly or indirectly (e.g. via water), is more likely with sheep faeces than with chicken since almost all chickens are housed, with faeces and litter collected and disposed (a significant proportion in the UK is actually incinerated). The case-control study did find that living in a rural area was associated with an increased likelihood of becoming infected by *C. coli* rather than *C. jejuni*, but there was no association with increasing density of sheep. This was also supported by the results from the case-case study that also failed to find an association with sheep density. These data provide little evidence that a large proportion of human cases are of sheep origin. A possible explanation is that there is a large diversity of STs in chicken, a number of which may exhibit a lower virulence in humans. All of the *C. coli* strains originating from sheep in the current study are also found in both chicken and humans. It may be that these strains are more likely to cause disease in humans. Indeed, the sheep-associated strains excreted into the environment could be a source for chicken colonisation which ultimately infects humans. Sheep have a *C. coli* prevalence of 11%, can shed up to 10^6^ CFU/g in their faeces [15], can be grazing nearby to broiler houses and only a small breakdown of biosecurity (e.g. via insects, rodents, contaminated drinking water, physical transfer via the soles of boots) can lead to contamination of the broiler flock. This explanation highlights one of the main disadvantages of human source attribution, in that it does not take account of the transmission pathway. It only compares the distribution of strains in the source reservoirs with the human host. That being said, the comparison of source attribution reservoirs to risk factors has been demonstrated to be biologically feasible for *C. jejuni* [18]. Further work is required to improve our understanding of the infection routes, sources and epidemiology of *C. coli* infections that are evidently different from *C. jejuni*. A case-control study, with associated genotyping could be conducted to include more proximate risk factors (e.g. contact with sheep, drinking from private water supplies, handling raw chicken etc.).

### Conclusions

The aetiology of human *C. coli* infections is similar in a number of respects to *C. jejuni* but there are important differences. There is an increased risk of *C. coli* infection in the older people, in people who live in rural areas and during the summer months. Public health together with national and international food safety agencies should take these differences into account when considering interventions to reduce the incidence of this gastrointestinal pathogen.

## Supporting Information

File S1 ***C. coli*** **and** ***C. jejuni*** **human clinical data including all 9 raw and transformed (for logistic regression) variables together with MLST sequence type.**
(XLSX)

File S2 **Host attribution scores for each** ***C. coli*** **ST.**
(XLSX)

File S3 **List of all isolates by ST and host.**
(XLSX)

File S4 **Cut-off attribution scores used to classify human clinical** ***C. coli*** **isolates as chicken or non-chicken strains.**
(XLSX)
